# Population genetic diversity and fitness in multiple environments

**DOI:** 10.1186/1471-2148-10-205

**Published:** 2010-07-07

**Authors:** Jeffrey A Markert, Denise M Champlin, Ruth Gutjahr-Gobell, Jason S Grear, Anne Kuhn, Thomas J McGreevy, Annette Roth, Mark J Bagley, Diane E Nacci

**Affiliations:** 1 Population Ecology Branch, Atlantic Ecology Division, U.S. Environmental Protection Agency, 27 Tarzwell Dr., Narragansett RI, USA; 2 Molecular Ecology Research Branch, Ecological Exposure Research Division, U.S. Environmental Protection Agency, 26 Martin Luther King Dr., Cincinnati OH 45268, USA; 3 Department of Natural Resources Science, Coastal Institute, University of Rhode Island, 1 Greenhouse Rd., Kingston RI 02881, USA; 4 c/o U.S. Geological Survey, San Diego Field Station, Western Ecology Research Center, 4165 Spruance Rd., San Diego, CA 92101, USA

## Abstract

**Background:**

When a large number of alleles are lost from a population, increases in individual homozygosity may reduce individual fitness through inbreeding depression. Modest losses of allelic diversity may also negatively impact long-term population viability by reducing the capacity of populations to adapt to altered environments. However, it is not clear how much genetic diversity within populations may be lost before populations are put at significant risk. Development of tools to evaluate this relationship would be a valuable contribution to conservation biology. To address these issues, we have created an experimental system that uses laboratory populations of an estuarine crustacean, *Americamysis bahia *with experimentally manipulated levels of genetic diversity. We created replicate cultures with five distinct levels of genetic diversity and monitored them for 16 weeks in both permissive (ambient seawater) and stressful conditions (diluted seawater). The relationship between molecular genetic diversity at presumptive neutral loci and population vulnerability was assessed by AFLP analysis.

**Results:**

Populations with very low genetic diversity demonstrated reduced fitness relative to high diversity populations even under permissive conditions. Population performance decreased in the stressful environment for all levels of genetic diversity relative to performance in the permissive environment. Twenty percent of the lowest diversity populations went extinct before the end of the study in permissive conditions, whereas 73% of the low diversity lines went extinct in the stressful environment. All high genetic diversity populations persisted for the duration of the study, although population sizes and reproduction were reduced under stressful environmental conditions. Levels of fitness varied more among replicate low diversity populations than among replicate populations with high genetic diversity. There was a significant correlation between AFLP diversity and population fitness overall; however, AFLP markers performed poorly at detecting modest but consequential losses of genetic diversity. High diversity lines in the stressful environment showed some evidence of relative improvement as the experiment progressed while the low diversity lines did not.

**Conclusions:**

The combined effects of reduced average fitness and increased variability contributed to increased extinction rates for very low diversity populations. More modest losses of genetic diversity resulted in measurable decreases in population fitness; AFLP markers did not always detect these losses. However when AFLP markers indicated lost genetic diversity, these losses were associated with reduced population fitness.

## Background

Decreased population genetic diversity can be associated with declines in population fitness (e.g., [1,2]). These declines are thought to involve components of the so called genetic 'extinction vortex', which directly ties losses in population genetic diversity to increased extinction risk [3]. These losses cause a decrease in individual fitness through the expression of inbreeding depression-like effects, further reducing the effective population size (*N_e_*) and leading to additional increases in the number of alleles that are alike by state within individuals [4]. The impact of increased individual homozygosity on individual fitness has been extensively documented in both laboratory, semi-natural, and natural settings [2,5-9]. The effects are especially strong in altered or degraded environments [10-12], although the genomic basis of heterozygosity-associated fitness differences and heterosis are still debated [13-16]. In addition to increasing individual homozygosity, lost population genetic diversity also reduces the adaptive potential of a population. For populations to persist over extended time-spans, they must have sufficient allelic resources to adjust to novel selective regimes. Forces ranging from invasive parasites and diseases to shifting climatic patterns ensure that environmental conditions will fluctuate temporally and spatially for all populations. Some species have shown a striking capacity to rapidly adapt to novel selective pressures [17,18] while others have not [19,20]. Because overall population diversity affects both short-term individual fitness and long-term population adaptive capacity, there is a need to develop an empirical quantitative understanding of the relationship between population genetic diversity and population viability.

Many laboratory models have demonstrated the large role of genetic diversity in increasing population fitness mediated through heterosis, particularly when inbreeding levels are high. In one classic example, Leberg [21] found that populations of mosquito-fish founded with siblings grew more slowly than those founded by unrelated individuals. In a subsequent experiment using non-relatives and experimentally manipulated levels of genetic diversity, Leberg [22] detected no evidence of a relationship between genetic diversity and population fitness. By manipulating *N_e _*while holding the census size constant over three generations in the annual plant *Clarkia pulchella*, Newman and Pilson [23] were able to demonstrate that populations with a small *N_e _*were more than twice as likely to go extinct as larger populations. Similarly, in a multi-generation experiment using houseflies, Bryant et al. [24] detected clear declines in relative fitness in low founder number populations and in repeatedly bottlenecked populations, even when the number of individuals subjected to a given bottleneck was relatively large.

Frankham et al. [25] developed a more direct method for measuring the effect of population genetic diversity on adaptive potential by steadily increasing the level of an environmental stressor (NaCl) every generation in laboratory *Drosophila *populations. In this study, both mildly bottlenecked and highly inbred populations showed a reduced ability to evolve tolerance to an environmental stressor relative to outbred populations.

In order to understand long-term population viability in a changing environment, experimental models that can build upon these results must be developed. Several published studies provide evidence that severely reduced genetic diversity can affect population fitness, but the impacts on population viability of modest (and perhaps more commonly occurring) reductions in genetic diversity are less well characterized. Further, many laboratory studies of evolutionary processes have relied on *Drosophila *or *Tribolium *(e.g., [8,25,26]). Both organisms have many experimental advantages, but their very high fecundities [27,28]--which can facilitate rapid rates of adaptation--make them poor models for vertebrate species with much lower reproductive rates. Laboratory models with lower fecundity may be more directly relevant to vertebrate conservation. Ideally, models of evolutionary genetics should also be able to disentangle the effects of population history and the effects of inbreeding from the adaptive potential represented by genetic diversity *per se*. To do this, they must also allow for fitness to be measured in multiple environments.

Here we present data from laboratory populations of the mysid shrimp (*Americamysis bahia*) a small crustacean native to estuaries along the US East coast [29]. This animal model has several experimental advantages that make it a valuable tool in evolutionary and conservation genetics. Because they are widely used in toxicological studies, optimal culture conditions and demographics are well characterized [30-32]. Time from conception to first mating is approximately three weeks at 25°C and 30 parts per thousand (ppt) salinity [31,33]. Mature females can produce a new brood every seven days and provide an unusually high level of brood care for an invertebrate; they incubate a small number of fertilized eggs in a marsupium for seven days, giving *A. bahia *a reproductive profile more similar to many birds and mammals than to other more fecund invertebrates. Owing to their estuarine habitat, *A. bahia *tolerate a wide range of salinities. In laboratory settings at 25°C, *A. bahia *cultures reproduce well in natural seawater with a salinity of 31 ppt NaCl, although they are reproductively viable in as little as 10 ppt NaCl [31]. In the wild, *A. bahia *have been collected in waters with salinity as low as 3 ppt, although some field surveys suggest they are uncommon below 9 ppt [34].

By simultaneously manipulating the selective environment and genetic diversity under controlled laboratory conditions with replication, we used *A. bahia *cultures to develop a more detailed understanding of the relationship between genetic diversity and population fitness in a changing environment. We also generated AFLP [35] genotypes for many of the populations to determine how well a typical molecular genetic fingerprint analysis predicts meaningful losses of genetic diversity. Our study goal was to develop a model system for quantifying the general relationship between genetic diversity and fitness in both permissive and stressful environments.

## Methods

### Collection of stock populations

*Americamysis bahia *were collected by dragging a fine-mesh net in shallow waters near Biloxi Beach, MS USA (N30.39351, W088.90123) and Navarre Beach, FL USA (N30.38964, W086.83050) during April 2005. Live animals were keyed out under dissecting microscopes at the US-EPA's Gulf Ecology Division in Gulf Breeze, FL USA. Approximately 50 individuals from each collection site were then transported to the US-EPA's Atlantic Ecology Division facilities in Narragansett, RI USA. Populations derived from each of the two collection sites were housed separately in four 80 L tanks with flow-through seawater maintained at 25°C and an ambient salinity of approximately 30 ppt. Animals were fed Selco enriched *Artemia ad lib *[36]. *Americamysis bahia *cultures grew quickly to more than 2000 individuals from each source.

### Generation of low diversity lines

Replicate lines with low genetic diversity were generated through a series of population bottlenecks. Individual lines were housed in 9.4 L tanks (environmental conditions as above). In late June 2006, 32 gravid females were selected from each of the two source populations and placed in separate tanks to become founders for 64 low diversity lines (parental generation). Following the release of young (F_1 _generation), the founding-females were removed, and their offspring were allowed to grow to maturity and breed for a period of three weeks. After this time, two gravid F_1 _females were selected from each line and remaining individuals were discarded. These F_1 _founders were removed after they had released their broods, producing the F_2 _generation. After the broods matured and became reproductively active, a single gravid F_2 _female was selected to found F_3 _and subsequent generations within each line. If the initial founding female was fertilized by a single male (a reasonable assumption given mysid reproductive biology), then pedigree based estimates suggest an average 31% decrease in heterozygosity ( = 0.3125). Alternatively, the 2-4-2 bottleneck represents a harmonic-mean effective population size of 2.4 individuals and a 50% decrease in heterozygosity relative to the starting populations [37]. Starting with the F_3 _generation, random mating was permitted within each line.

### Generation of high diversity lines

*Dihybrid (2x) Lines *- The viable low diversity lines generated through bottlenecks (above) were designated '1x', and represented the lowest level of genetic diversity in our study. Randomly chosen sets of 1x lines were crossed to generate higher diversity levels. Briefly, to generate 2x lines three gravid females were selected from one of the randomly selected low diversity lines. Their offspring were discarded once they were released, and these mature, now non-gravid females were randomly paired with two males from another low diversity line. Crosses were performed randomly yielding four Navarre × Navarre lines, three Biloxi × Biloxi lines and eight Navarre × Biloxi lines. As the 1x parents of the 2x lines were independently created from the base populations, the inbreeding coefficent for 2x lines was zero ( = 0).

*6*x, *8*x *and Admixed Lines - *Populations containing the genetic equivalent of either six or eight 1x lines were created as the main experiment was established (see below). We created these higher diversity levels by combining individuals from different dihybrid lines. The founding number for each population was 12 individuals, so 6x lines were founded by randomly choosing four individuals from three 2x lines. Similarly, 8x lines were founded by choosing three individuals from each of four unique 2x lines. The founding 2x lines were chosen randomly, with the constraint that any ancestral 1x population could be used only one time within a 6x or 8x population ( = 0). Lines with the highest level of diversity, "Admixed (AMX)" were obtained by drawing six individuals each from the Biloxi and Navarre stock populations as founders. We chose to combine individuals from the stock populations to maximize the level of allelic diversity in the highest diversity populations. Space constraints did not permit us to include Biloxi and Navarre stock populations individually, however a series of pilot experiments (not shown) did not suggest any performance differences between these founding populations.

### Salinity and culture

A pilot study demonstrated that reproductive rates for *A. bahia *were similar in ambient seawater and at 10 ppt salinity (data not shown). When the salinity was reduced to 7 ppt, reproduction ceased. Based on this preliminary data, together with published findings [31], and our expectation that low genetic diversity populations would be more sensitive to environmental stress, we chose 9 ppt salinity as the target level of novel environmental stress.

Experimental populations were housed in 9.4 L tanks with precisely controlled salinities, light cycles, and temperatures. Both ambient seawater and seawater diluted with dechlorinated tap water were available via a flow through system, and we ran water through the tanks each day for one hour in the morning and one hour in the evening to ensure precise control of salinities. At the observed flow rate, this was sufficient for more than one complete exchange daily. Tanks were kept in two water tables to ensure uniform temperatures between tables and replicates. Tanks were moved within and between tables weekly to further reduce the potential for position effects. Lights were on a 12:12 light:dark cycle with gradual transitions to simulate natural conditions. Salinity was measured using a Hach meter Model 60 d. Salinity and temperature were measured daily in a randomly selected 10% of tanks. The mean temperature for all measured tanks was 25.3°C (± 0.03 S.E.). Low salinity tanks were maintained at a mean of 9.4 (± 0.07) ppt. Normal seawater tanks had an average of 29.4 (± 0.50) ppt. Animals were fed *ad libidum *with Selco-enriched [36]*Artemia *(Aquafauna Biomarine, Hawthorne, CA USA).

### Experimental design

#### Phase 1 - population establishment and expansion

Experimental aquariums were established as matched pairs, one serving as a control (permissive environment) and one subjected to low salinity (stressful environment). Experimental populations were founded with 12 individuals (see above) and these were allowed to breed and expand for three weeks in a permissive environment (~30 ppt salinity).

#### Phase 2 -- chronic low salinity stress

After this initial census, designated experimental populations were subjected to a stressful environment by gradually reducing the salinity to 9 ppt over the course of four days. Salinity was maintained at this level thereafter. The remaining control tanks were maintained with normal seawater. During the experimental period, a weekly census was conducted in which all individuals were counted and the presence of neonates (animals < 7 days old) was noted.

Fifteen pairs of low diversity (1x) lines were established. We intended to establish these cultures from 15 independently bottlenecked lines, however one of the designated lines went extinct before the start of the experiment, so one of the surviving lines was used twice. Fifteen independent pairs of 2x cultures were also established. Higher diversity levels (6x, 8x and Admixed) were replicated 10 times. The entire experiment contained 120 tanks. A summary of the experimental design is shown in Table 1.

**Table 1:** Basic experimental design and levels of replication

Nominal Diversity	Nominal Diversity	Number of Replicates	Environment
**1x**	0	15	Low Salinity
		
		15	High Salinity

**2x**	0.5	15	Low Salinity
		
		15	High Salinity

**6x**	0.833	10	Low Salinity
		
		10	High Salinity

**8x**	0.875	10	Low Salinity
		
		10	High Salinity

**Admixed**	-	10	Low Salinity
		
		10	High Salinity

Relative diversity is defined here as the expected heterozygosity assuming that the 1x lines were homozygous at all loci.

At the end of the 14-week survey period, surviving individuals were preserved in 100% ethanol from each tank for molecular analysis.

### Adaptation over time

To estimate the response to selection of each nominal genetic diversity level over the course of the experiment, population sizes in the stressful and permissive environments were compared three weeks (~1 full reproductive cycle) after the environmental stress was introduced and at the end of the experiment (~3 reproductive cycles later).

### Genetic analysis

AFLP genotypes generated from surviving control populations at the end of the experiment were used as a measure of starting genomic diversity for each diversity level. It was not possible to genotype the founding populations at the beginning of the experiment because the low diversity stock lines had only a modest number of individuals, and most of these were required to found the experimental populations. For the lowest diversity lines, the harmonic mean population size was 33.8 individuals, which suggests that the populations would have lost about 2% of their heterozygosity due to genetic drift each generation. In the highest diversity populations, *N_e _*was estimated to be 110.6 individuals, consistent with a decline in neutral locus heterozygosity of less than 1% per mysid generation. Some lines were excluded from the molecular analysis because fewer than 10 individuals were available.

Ten individuals were randomly chosen from each line to estimate genetic diversity. DNA was extracted from whole *A. bahia *using DNeasy^® ^Blood and Tissue kit (Qiagen, Valencia, CA, USA). The manufacture's instructions were followed except that we heated the elution Buffer AE to 70°C for 10 minutes and incubated the sample with Buffer AE for five minutes at room temperature before eluting each DNA sample. Genomic DNA was quantified using Quant-iT™ PicoGreen^® ^dsDNA Assay Kit (Invitrogen, Carlsbad, CA, USA) with a Synergy™ HT Multi-Mode Microplate Reader (BioTek, Winooski, VT, USA).

AFLP analysis followed the procedure of Vos et al. [35], modified to accommodate fluorescent visualization and using the restriction enzyme pair EcoRI/PstI [41]. Total genomic DNA (75 -- 200 ng) was simultaneously digested and ligated in a 15 μl reaction that included 5 units each of EcoRI, PstI, and T4 DNA ligase (New England Biolabs), 30 pmoles of each EcoRI and PstI double-stranded DNA adaptor [see [41]], 50 ng/ul BSA, and 50 mM NaCl in T4 Ligase buffer (New England Biolabs). Following complete digestion and ligation at room temperature, products were diluted ten-fold into 10 mM Tris pH 7.6, 0.1 mM EDTA.

Initial PCR enrichment of a subset of fragments (pre-amplification) used 5 μl of the diluted digestion-ligation product as template: 0.5 μM of the EcoRI + A/PstI + C primers (IDT, Coralville, IA) and 0.25 U Taq DNA polymerase (Invitrogen) in 20 μl of 20 mM Tris-HCl (pH 8.4), 50 mM KCl, 0.2 mM each dNTP, and 1.5 mM MgCl_2_. PCR conditions were 2 min at 74°C; 24 cycles of 94°C for 30 sec, 56°C for 30 sec; 72°C for 1 min; followed by 30 sec at 72°C. The pre-amplification product was then diluted ten-fold with 10 mM Tris pH 7.6, 0.1 mM EDTA buffer.

Selective amplification reactions were similar to pre-amplifications, with 3 μl of diluted pre-amplification product used as template and substituting 50 pM of the appropriate FAM--labeled EcoRI + 3/250 pM PstI + 2 selective AFLP primers. Three selective primer combinations were used on all samples: EcoRI + ACT--PstI + CT; EcoRI + AGG--PstI + CA; and EcoRI + ATG--PstI + CT. PCR conditions were 2 min at 94°C, 12 cycles of 20 sec at 94°C, 30 sec at 66°C dropping 1°C per cycle, 1 min at 72°C; then 20 cycles of 20 sec at 94°C, 30 sec at 56°C, 1 min at 72°C; followed by 30 min at 72°C. AFLP genotypes were electrophoresed and visualized with an ABI 3730 DNA analyzer.

Bins within the range of 100 to 500 bp [38] were generated for the amplified fragments using GeneMarker^® ^version 1.6 (SoftGenetics LLC^®^, State College, PA, USA). We manually checked the quality of each AFLP fingerprint and bin using the method described by Whitlock et al. [39] with slight modifications. We removed samples that produced an AFLP fingerprint with less than 20 peaks within the target size range and restricted our analyses to fragments with relative florescence units greater than 100 to reduce background noise. We visually checked the automatically created bins to ensure the bin was centered on the distribution of peaks within the bin and removed bins that had AFLP fragments that differed in size by more than 1 bp. We also deleted bins with fragment-length distributions that overlapped with adjacent bins to reduce the occurrence of homoplasy [38,40]. The number of initial bins for the three sets of restriction enzymes ranged from 63 to 76 each. We developed an R http://www.R-project.org/ script to convert the raw peak intensity data output from GeneMarker to a format compatible for AFLPScore version 1.3 [40]. We scored our raw AFLP data using AFLPScore, normalized our data to the median, filtered our data with a locus selection threshold, and used a relative genotype calling threshold. We tested a range of locus (100 to 1000 bp) and genotype thresholds (1 to 120%) and selected the pair of values that simultaneously minimized the mismatch error rate, minimized the probability of misscoring a presence allele (ε_1.0 _error rate), and maximized the number of loci retained. We included all pairwise comparisons for the samples that had greater than two replicates in our mismatch analysis. We generated AFLP genotypes for each restriction enzyme pair with the optimized locus selection and genotype thresholds using AFLPScore.

The locus selection threshold was 1000 bp and the genotype threshold was 10% for each restriction enzyme pair. The average mismatch error rate for the three restriction enzyme pairs was 8.3504 ± 1.7367 (S.D.) and the average ε_1.0 _error rate was 19.484 ± 2.3992. Following the intensive screening and quality control process, 59 loci (bins) were available to estimate genetic diversity. AFLP based estimates of genetic diversity were calculated using AFLP-Surv v1.0 [38]. AFLP based estimates of genetic diversity were calculated as either the fraction of polymorphic loci within the sample (PLP) or the heterozygosity analogue (H_j_) [41].

### Statistical analyses

Three different indices of population fitness were evaluated: 1) the number of individuals in the *Last Census *(LC), 2) the *Median Population Size *(MPS) using data from all 13 censuses for each experimental tank and 3) the *Reproductive Index *(RI), which was calculated as the number of weeks in which reproduction was observed divided by the total number of weeks that the population survived for each population.

Statistical relationships among fitness, genetic diversity (treating levels 1x, 2x, 6x, 8x, and Admixed as ordinal categorical data), and environmental stress were evaluated using general linear models. All calculations were performed using either JMP 7.0 or SAS 8.0 (SAS institute, Cary NC).

The genetic load of the inbred 1x was estimated relative to the outcrossed AMX lines using the methods of Morton and Crow [42]. Genetic load was not estimated during the creation of the inbred lines because reference populations required for the calculation were not established due to space limitations.

## Results

### Genetic Load and Extinction of Founder Lines

A substantial proportion of the bottlenecked lines either failed to thrive or did not survive long enough to be used in the main experiment. Of the 64 lines initiated, only 14 achieved a population size of at least 26 individuals, the predetermined threshold deemed sufficient to generate dihybrid lines and maintain the 1x lines.

Genetic load within the main experiment was estimated to be higher in the stressful environment for all three fitness indices. Using LC, the number of lethal equivalent loci in the permissive environment was 2.87 compared to 10.97 in the stressful environment. Lethal equivalents for MPS were 3.45 in the permissive environment and 6.71 in the stressful environment. For RI, in a permissive environment we estimate that there were 1.06 lethal equivalents compared to 3.19 in the stressful environment.

### Molecular estimates of genetic diversity

After screening, a total of 59 AFLP markers were available for analysis. Average PLP for the 1x lines was 35.6 ± 7.3 (S.D.) and average H_j _was 0.14 ± 0.05. In comparison, AMX lines had an average PLP of 52.1 ± 4.3 and an average H_j _of 0.19 ± 0.02. Nominal genetic diversity explained a moderate amount of variation in AFLP diversity estimates (PLP Spearman's ρ = 0.67, p < 0.0001; H_j _Spearman's ρ = 0.44, p = 0.0043). In post-hoc tests, neither estimator was effective at differentiating among the three highest genetic diversity treatments; however the 1x, 2x and higher diversity lines were distinguishable from each other when PLP was used to estimate genetic diversity (Table 2).

**Table 2:** Estimates of average neutral locus genetic diversity

Nominal Diversity	Avg. PLP	Avg. H_j_
**1x**	35.6 + 7.3^A^	0.14 + 0.05^A^

**2x**	43.1 + 9.2^B^	0.16 + 0.03^B^

**6x**	49.2 + 7.1^C^	0.19 + 0.02^C^

**8x**	47.8 + 5.1^C^	0.18 + 0.03^B,C^

**Admixed**	52.1 + 4.6^C^	0.19 + 0.02^C^

Average ± 1 S.D. for the percentage of polymorphic loci (PLP) and the heterozygosity analogue (H_j_) for the multilocus AFLP genotypes are provided. Letters unite groups that are not statistically distinguishable using Tukey's HSD.

### Population growth in permissive conditions

**Figure 1:**
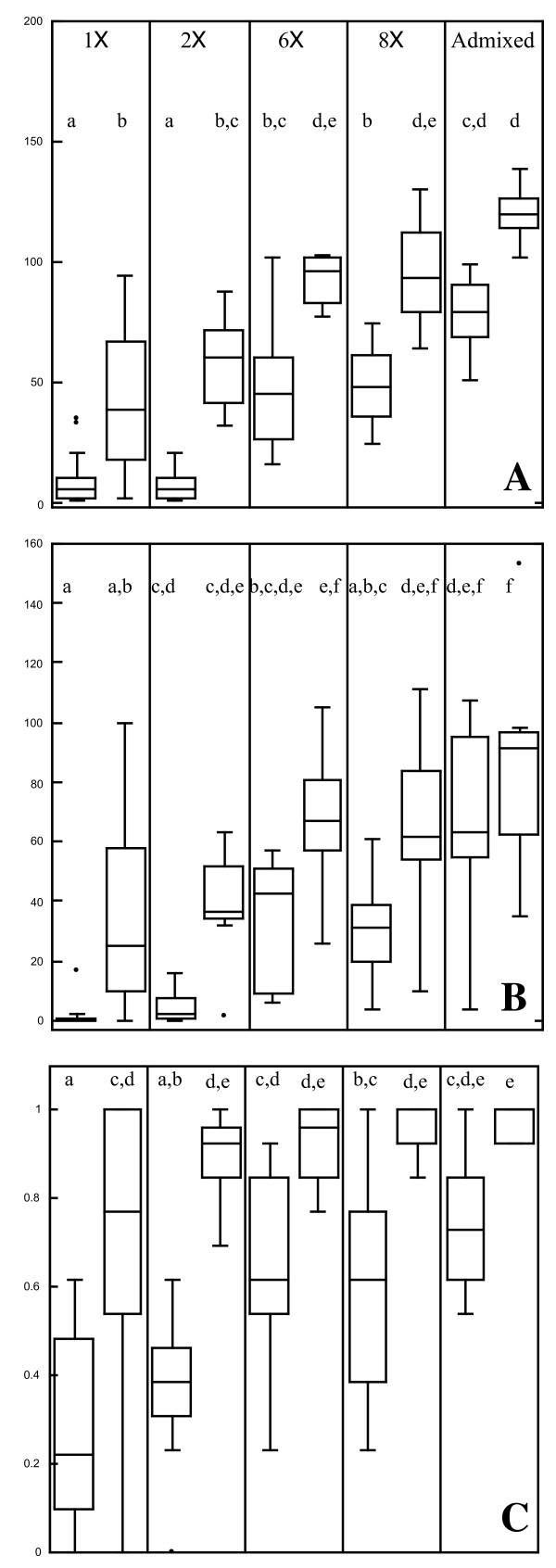
**Population fitness, estimated with Median Population Size (A), Last Census size (B), and Reproductive Index (C)**. Paired box plots define the median and middle two quantiles in stressful (left) and permissive environments (right). Lower case letters unite groups that are not statistically distinguishable using post-hoc tests (Tukey's HSD) at α = 0.05.

Abundance after three weeks of culture under permissive conditions (Phase 1) was significantly correlated with nominal genetic diversity level (Spearman's ρ = 0.68, p < 0.0001, Table 3). Final population sizes increased from 12 individuals at the start of the experiment to an average of 18.6 individuals in the low diversity lines (1x) and to 79.3 individuals in the highest diversity populations (AMX). All treatments differed from each other, except 6x and 8x. Variance was unequal among treatments (p = 0.0244) with the coefficient of variation inversely related to genetic diversity (Table 3, Figure 1).

**Table 3:** Averages and coefficients of variation for each experimental treatment

Diversity Level	Environment	*NI*		*MPS*		*RI *		*LC*		% Extinct	*TTE*
				
		**Avg**.	**C.V**.	**Avg**.	**C.V**.	**Avg**.	**C.V**.	**Avg**.	**C.V**.		
**1x**	**Permissive**	6.6	1.59	42.4	0.67	0.69	0.49	34.3	0.91	20	7
	**Stressful**	-	-	9.5	1.07	0.27	0.77	2.5	2.44	73	9

**2x**	**Permissive**	19.7	0.77	60.2	0.31	0.90	0.09	44.5	0.42	-	-
	**Stressful**	-	-	19.0	0.64	0.38	0.44	6.2	1.27	7	11

**6x**	**Permissive**	33.7	0.49	95.3	0.19	0.93	0.09	66.3	0.37	-	-
	**Stressful**	-	-	49.7	0.50	0.68	0.34	33.6	0.58	-	-

**8x**	**Permissive**	33.3	0.43	94.9	0.22	0.94	0.06	63.6	0.49	-	-
	**Stressful**	-	-	47.4	0.38	0.57	0.41	28.4	0.63	-	-

**Admixed**	**Permissive**	67.3	0.28	123.2	0.20	0.97	0.04	84.4	0.39	-	-
	**Stressful**	-	-	77.4	0.21	0.73	0.22	65.6	0.47	-	-

The Net Increase (*NI*) was calculated after three weeks in permissive conditions. *MPS *is the median population size calculated using weekly census data from 13 post stress weekly censuses. *RI *is the Reproductive Index: the fraction of census weeks in which reproduction was observed. *LC *is the population size at the Last Census. *TTE *is the median Time To Extinction among lines that went extinct.

AFLP diversity estimated as PLP explained a modest percentage of the variation in abundance after three weeks in permissive conditions (adjusted R^2 ^= 0.24, p < 0.0001). AFLP diversity estimated as H_j _explained less but still significant abundance variation (adjusted R^2 ^= 0.16, p < 0.0001).

### Population fitness, environmental stress and genetic diversity

A model including genetic diversity and environmental stress explained much of variation in MPS during the chronic low salinity experiment (Phase 2) (adjusted R^2 ^= 0.74, p < 0.0001). Both factors contributed strongly to the relationship (environment F = 127.8, p < 0.0001; diversity F = 53.3, p < 0.0001). Treatment means ranged from 9.7 individuals (low salinity, 1X) to 123.2 individuals (normal salinity, AMX). There was no significant interaction between salinity stress and genetic diversity level (F = 0.59, p = 0.6693) (Figure 1).

An additional model that included the results of the first census (Phase 1, pre-stress) as a covariate also explained much of the variation in MPS (Adj R^2 ^= 0.78, p < 0.0001). Abundance at initiation of experimental treatments was a significant covariate (F = 16.5, p < 0.0001). In this more complex model, there was a significant interaction between initial abundance and genetic diversity level (F = 4.0, p = 0.0043), but no interaction between environment and genetic diversity (F = 0.78, p = 0.536). Both nominal diversity level (F = 13.1, p < 0.0001) and environment (F = 22.3, p < 0.0001) were significant individually.

The last census sizes ranged from a mean of 2.5 individuals (low salinity, 1x) to 84.4 individuals (normal salinity, AMX). A model including nominal genetic diversity and environmental stress explained 53% of the observed variation in LC (p < 0.0001). Genetic diversity (F = 50.8, p < 0.001) and environmental stress (F = 21.4, p < 0.001) were both significant, but the interaction term was not (F = 0.54, p = 0.71). An expanded model for LC that included the results of the first census (Phase 1, pre-stress) as a covariate explained no additional variation in LC (Adj R^2 ^= 0.53, p < 0.0001), and the covariate was marginally insignificant (F = 3.45, p < 0.0658).

Environmental stress and genetic diversity explained much of the variation in RI (adjusted R^2 ^= 0.58, p < 0.0001). Both factors were statistically significant (stress F = 95.4, p < 0.0001; genetic diversity F = 14.3, p < 0.0001) with a marginally insignificant interaction between these two factors (F = 2.18, p = 0.0751).

**Figure 2:**
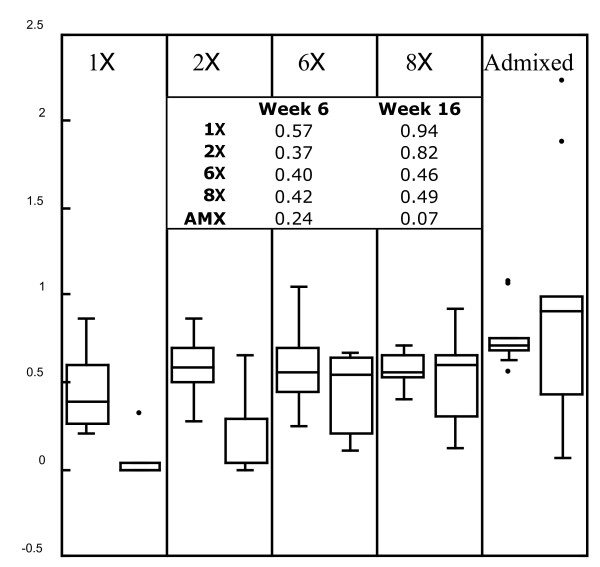
**Ratios of census sizes in the stressful environment to those in the permissive environments for each diversity class after three weeks in the selective environment (left box plot) and at the end of the experiment (right box plot)**. The box plots enclose the central two quantiles and show the group medians. Only the 1x and 2x treatments are significantly distinguishable using the Wilcoxon signed rank test. The inset shows the average percent decline in census size in the stressed populations relative to the control populations at the end of the experiment.

In these analyses, variance was unequal among treatments, and remained unequal despite attempted transformations. Variance was higher among low diversity populations and under stressed conditions (Figure 2). Variation (expressed as the coefficient of variation) in all three fitness proxies is summarized in Table 3, and the distribution of individual replicate values is shown in Figure 1. The most likely effect of unequal variances in these analysis is an increase in Type I error, which could be compensated for by reducing α by half to 0.025 [43]. All effects that were previously found to be significant remain significant under this more stringent criterion.

### Population fitness, environmental stress and molecular diversity

The effects of AFLP diversity (estimated for each individual replicate using either PLP or H_j_) and environmental stress were evaluated for three different fitness indices: MPS, LC, and RI.

A significant portion of the variation in MPS is explained by a model incorporating AFLP diversity measured as PLP and environmental stress (adjusted R^2 ^of 0.53, p < 0.0001). Both variables were statistically significant (stress F = 59.1, p < 0.0001; PLP F = 32.9, p < 0.0001), and there was no significant interaction between the two terms (F = 0.04, p = 0.83). Similar results were obtained when H_j _was substituted for PLP (adjusted R^2 ^= 0.51, p < 0.001; stress F = 56.1, p < 0.0001; H_j _F = 27.2, p < 0.0001; stress*H_j _F = 0.26, p = 0.61).

Models evaluating the effect of AFLP diversity and environmental stress on LC were also significant overall (adjusted R^2 ^= 0.37, p < 0.0001 using PLP, adjusted R^2 ^= 0.38, p < 0.0001 using H_j_). There was no interaction between genetic diversity and stress in either model using PLP (PLP F = 11.8, p < 0.0001; stress F = 36.8, p = 0.001; stress*PLP F = 0.58, p = 0.45) or H_j _(H_j _F = 13.5, p < 0.0004; stress F = 37.2, p < 0.0001; stress*H_j _F = 0.0009, p = 0.97).

Similarly, both PLP and H_j _explained a significant fraction of the variation in RI (PLP Adj R^2 ^= 0.53, environment F = 75.6, p < 0.0001, PLP F = 15.6, p = 0.0002), (H_j _Adj R^2 ^= 0.52, environment F = 73.9 p < 0.0001, H_j _F = 14.0, p = 0.0002). Neither genetic diversity estimator had a significant interaction with environmental stress.

### Observed population extinctions

Population extinctions were rare during the course of the study, and were confined to the low diversity populations (Table 3). Three out of 15 1x populations went extinct under permissive conditions. Median time to extinction for these populations was seven weeks. By contrast, 11 of 15 1x populations went extinct under stressful conditions, with a median extinction time of nine weeks. Only a single 2x population went extinct in the low salinity treatment at 11 weeks.

AFLP data were available for nine of the 15 pairs in the lowest diversity 1x treatment. The remaining six pairs could not be surveyed due to extinction or low survivor numbers in the control line. The lines that went extinct had a mean PLP of 32.5 compared to 39.4 for the surviving lines, although this difference was not significant (p = 0.17). H_j _in extinct lines was 0.11 and 0.18 in surviving lines, and the difference was statistically significant (p = 0.014) (Table 2).

### Adaptation over time

After three weeks exposure to low salinity (Week 6 of the experiment), the average census size for 1x populations in this stressful environment was 57% smaller than those in the high salinity control environment, while the high diversity AMX lines were, on average, 24% smaller in the stressful environment relative to their controls. At the end of the experiment (Week 16), census sizes for 1x stressed populations were 94% smaller than their controls on average, whereas average AMX populations reared in low salinity was only 7% smaller than their controls. The relative decline in census size of the salinity stressed 1x populations was partly driven by the extinct lines; however, when these were excluded the net decline relative to the control population was still 83% (Figure 2).

## Discussion

The experimental results presented here indicate that the *Americamysis bahia *system for generating defined levels of genetic diversity with a high level of replication is a useful tool for addressing empirical questions in conservation genetics. The results from this initial experiment measure: 1) the relative performance of high and low diversity populations in both good and bad environments; 2) the power of AFLP markers to detect meaningful losses of genetic diversity; 3) the magnitude of genetic load in both good and bad environments; 4) the potential utility of genetic rescue and heterosis; and 5) the relative potential for adaptation to novel environments.

### Reduced diversity and population fitness

In this simplified laboratory environment, lower population genetic diversity was associated with lower population fitness, although this decrease was not always statistically significant in all post-hoc tests. As expected, average population fitness in the stressful environment was always lower than fitness in the permissive environment for a given level of genetic diversity.

Interestingly, none of the interaction terms between nominal genetic diversity and environmental stress were significant for any of our three fitness indices although the interaction was significant for RI. This may indicate that factors interact less in our laboratory setting than might be expected in a more complex natural environment, but we cannot rule out the possibility of insufficient statistical power. Power analysis could potentially address this issue, however we lack a non-arbitrary estimate of the magnitude of a meaningful effect [44]. Similarly, we did not detect an interaction between either of the AFLP based diversity estimates and the environment when estimating fitness. This particular analysis is complicated by the fact that extinctions in some of the 1x control lines reduced the number of lines available with very low diversity.

A modest amount of neutral locus genetic diversity (as estimated with AFLP genotypes) was explained by nominal diversity level. The overall relationship was in the expected direction; however, post hoc tests (Table 2) reveal that estimates of both PLP and H_j _based on our final set of 59 screened AFLP markers do not reliably detect differences between the three highest nominal diversity levels. Similarly, both estimators explain only a modest amount of the variation in the three fitness indices. Despite the lack of a detectable molecular genetic difference, the observed mean fitness was always lower in 8x populations than in AMX populations in the stressful environment and for two of the three fitness proxies in the permissive environment. Post-hoc tests showed these differences were statistically significant for two of the three proxies in the stressful environment (Figure 1).

In this simplified experimental environment, AFLP markers detected large decreases in genetic diversity but missed more modest but ecologically meaningful losses. This may have important implications for the application of AFLP genotypes. While AFLP markers lack the power to detect all meaningful losses of genetic diversity, these markers are unlikely to cause false positives; when detectable losses in AFLP diversity occur, our data suggest they signal a serious decline in population viability.

### Inbreeding, Genetic load and Hybrid Rescue

The clearest evidence for the effects of inbreeding on *A. bahia *populations was obtained before the formal experiment started. In order to generate the 1x lines used in this study, we started with 64 founding lines. Fully three quarters of these lines failed to generate the 26 individuals that were required to found the experimental lines after several months in culture. Some early losses may also be due to demographic stochasticity--initial brood sizes are small in young mysid females. However many lines that survived failed to thrive during more than four months under permissive conditions. Thus, inbreeding effects were a major determinant of the number and types of lines available for our main experiment making it necessary to construct experimental populations using only the modest number of lines that were *most resistant *to inbreeding depression. This result is typical of animals with large, panmictic populations [24,45].

From a conservation genetics perspective, it is important to understand the population level consequences of individual inbreeding depression (or the approximate opposite, heterosis). It has been repeatedly noted that the impact of individual inbreeding depression varies with environment [46,47], and the negative effects of high levels of inbreeding may be masked by permissive environments or when a direct comparison with outbred individuals is not possible [6,12,48]. In experimental settings, inbreeding depression is usually, but not universally, stronger in stressful environments [46]. In the mysid experimental system, estimates of the genetic load in both environments suggest that while inbreeding depression is expressed for all fitness metrics in the permissive environment, the effects are far more pronounced in the stressful environment. We note that owing to experimental constraints, this estimate of genetic load is applicable only to the main experiment and does not necessarily reflect genetic load within natural populations.

Because we constructed our higher diversity populations by combining different numbers of low diversity lines, our study may be viewed as a series of replicated 'genetic rescue'[49] experiments (albeit with very high immigration rates, comparable to [45]). Population fitness was substantially improved when two or more 1x lines were combined, and in almost all cases, the 'rescue' was successful. Only a single 2x population went extinct in the stressful environment. Within our system, nominal genetic diversity was an important predictor of population fitness for most levels of genetic diversity. In both environments and for all three of the fitness proxies, the 2x lines performed better on average than the 1x lines, and the 6x lines performed better than the 2x lines. The difference was not always statistically significant in post-hoc comparisons for each fitness index at each level (Figure 1), but the relative performance was as expected. Further, the high diversity AMX populations were generally more fit than any of the lower diversity populations.

We did not detect a statistically significant difference between the 6x and 8x populations in any of the fitness assays or by using molecular markers. We note that the best performing 8x were superior to the best performing 6x populations, however the worst performing 8x populations were inferior to the worst performing 6x populations. Because the 8X lines were founded with only three individuals from each of four founding 2x lines, it is possible that some of the founding lines did not establish themselves in some 8x populations. In any case, genetic diversity levels in these two classes are expected to be quite similar. Even for a locus that is fixed for alternate alleles in the 1x populations, expected heterozygosity of 6x and 8x populations would only differ by 4% on average (H = 0.833 and 0.875, respectively [50]). The actual heterozygosity difference is likely under 2% since 1x lines would have experienced only a 30% to 50% reduction in heterozygosity relative to the founding stock populations.

### Diversity, selection and adaptation

Many studies have focused on the individual fitness consequences of inbreeding in benign and stressful environments due to inbreeding depression effects [46] but this is only one way that genetic diversity affects extinction risk. It also is important to determine the consequences of reduced genetic diversity for the capacity of the population to adapt to a novel environment. Even modest losses of genetic diversity may result in a reduced ability to adapt to environmental change, yet the short-term impact of such losses may be minimal if populations are maintained in stable environments or if the loss does not cause detectable inbreeding depression-like effects. The long-term impact of moderate losses on population persistence can best be measured by estimating generational changes in population fitness in multiple environments. The mysid experimental system demonstrates that both population fitness and inter-population variability are influenced by genetic diversity, and that both fitness and variability are influenced by environmental stress.

To assess the strength of selection in the stressful environment, we calculated the ratio of populations in the stressful environment to those in the permissive environment three weeks (~1 mysid generation) after the stressful environment was introduced. We hypothesized that the relative proportions should be similar at both time points if inbreeding and heterosis are influencing the relationship, but that when adaptation has occurred, population sizes in the stressful and permissive environments will grow more similar over time. We found that after three weeks of selection the 1x population sizes in the stressful environment were 57% smaller than those in the permissive environment, while the AMX population sizes were only 24% smaller in the stressful environment. These declines represent the selection pressure imposed by the stressful environment. After 10 more weeks of selection, the AMX population sizes in the stressful environment were only 7% lower than those in the permissive environment while the 1x population sizes were 94% lower (Figure 2). Therefore, the low diversity populations did poorly in the stressful environment early in the experiment and grew progressively worse as the experiment proceeded. By contrast, the high diversity populations were relatively less disadvantaged early on and even showed some improvement by the end of the experiment. In the AMX lines the level of improvement did not rise to statistical significance; however, the trend was consistent with the one predicted by evolutionary adaptation (and some stressed populations even outperformed their matched controls), suggesting that simple heterosis may not be the only force operating in populations with high genetic diversity. However, these results should be interpreted with some caution as the high diversity populations may have been close to the carrying capacity of the habitat in both normal and low salinity environments.

In our mysid data set, nominal genetic diversity was an important predictor of variability between populations within an environmental treatment with lower diversity populations having more inter-population variability than higher diversity populations. Population size (either median or final) was also notably lower in low genetic diversity populations, so a much higher fraction of low diversity lines are likely to fall below the minimum number of individuals required to maintain population viability [51]. In general, temporal variation in abundance within a single population is expected to increase the chances of population loss [51,52], so these results indicate that genetic diversity is an important component of extinction risk.

## Conclusions

Using the mysid experimental system, we found that: 1) reduced population genetic diversity reduces population fitness in both permissive and stressful environments; 2) even some modest reductions in genetic diversity can reduce the value of some fitness measures, especially in stressful environments; 3) environmental stress and genetic diversity appear to independently influence population fitness; and 4) AFLP genotypes detected large reductions in population genetic diversity, but did not reliably detect modest reductions in genetic diversity that may influence population fitness. Therefore, many more AFLP loci than are commonly used would be necessary to detect these losses. However when genetic diversity losses are detected using a moderate number of AFLP loci, they are likely to be ecologically important. We also found that: 5) low diversity populations show more inter-population variability than high diversity populations for most estimates of population fitness; and 6) high diversity populations showed some capacity to adapt to the stressful environment, but low diversity populations did not.

In natural populations the relationship between population fitness and genetic diversity will depend on specifics of the environment and the organism. Genetic diversity may not always enable populations to persist, but a lack of diversity essentially guarantees that adaptation to altered environments will not occur. Despite the importance of diversity for population survival, our understanding of the relationship between diversity and long-term population viability is limited. Studies in simplified laboratory environments, such as the one described here, can be used to determine a baseline for the relationship between diversity and population risk under the best possible conditions (i.e., with the least environmental variation) and provide an important way to assess molecular tools that are potentially useful in conservation biology.

## Abbreviations

AMX: Admixed lines; H_j _: Heterozygosity estimate derived from dominant molecular markers; LC: The Last Census; the number of individuals in an aquarium at the end of the experiment; MPS: Median Population Size for a single line over the course of the experiment; NI: Net Increase in population size after three weeks in permissive conditions; PLP: Proportion of Loci Polymorphic; the fraction of AFLP bands that vary within an experimental populations; RI: Reproductive Index; the fraction of census weeks in which neonates were observed; TTE: Time To Extinction.

## Authors' contributions

JAM, DEN and MJB took the lead in designing overall experiment, while AK, JSG and DMC provided critical insights into key sections of the design. DMC, AK and JAM also conducted an extensive series of pilot studies that made this project possible. RG-G designed methods that allowed us to precisely maintain the experimental environment. TJM-optimized objective AFLP scoring parameters, produced AFLP genetic diversity estimates, and wrote the AFLP scoring section. AR managed the collection of molecular data and wrote the molecular section of the AFLP methods. All Narragansett based authors participated in weekly censuses and daily culture activities. All authors contributed to the writing of the paper and have read and approved the final manuscript.
